# Determinants of a sense of insecurity among home-dwelling older people

**DOI:** 10.1177/14034948221131419

**Published:** 2022-10-21

**Authors:** Mia T. Knuutila, Tuuli E. Lehti, Helena Karppinen, Hannu Kautiainen, Timo E. Strandberg, Hannareeta Öhman, Niina M. Savikko, Anu H. Jansson, Kaisu H. Pitkälä

**Affiliations:** 1 Social Services and Health Care, City of Helsinki, Finland; 2 Department of General Practice and Primary Health Care, University of Helsinki, Finland; 3 Unit of Primary Health Care, Helsinki University Hospital, Finland; 4 Clinics of Internal Medicine and Geriatrics, Helsinki University Hospital, Finland; 5 Department of Internal Medicine and Geriatrics, University of Helsinki, Finland; 6 City of Espoo, Elderly Care, Finland; 7 The Finnish Association for the Welfare of Older People, Finland

**Keywords:** Sense of insecurity, aged, Oldest old, loneliness, ageism

## Abstract

*Aims:* A sense of insecurity may have an impact on older people’s well-being and their courage to engage actively in meaningful activities. Studies on a sense of insecurity among older people are scarce. The aim of this study was to determine the extent to which home-dwelling older adults perceive their life as being insecure and how a sense of insecurity is associated with their health, functional status, active social engagement, well-being and perceptions of the societal treatment of older people. *Methods:* This study is part of the Helsinki Aging Study, a cohort study ongoing since 1989. Data were collected using a postal questionnaire that was mailed in 2019 to a random sample of home-dwelling older people ⩾75 years of age living in Helsinki (*N*=2917; response rate 74%). The questionnaire inquired about the respondents’ sense of security/insecurity, and they were subcategorised into those feeling secure and those feeling insecure based on their answers. *Results:* Seven per cent of respondents felt insecure in their lives. In a stepwise logistic regression analysis, loneliness, living alone and perceived poor societal treatment of older people were associated with a sense of insecurity, while having good self-rated health, having children and meeting friends at least weekly were associated with lower odds of insecurity. ***Conclusions:* Our findings highlight the importance of recognising and combating loneliness, social isolation and societal ageism in order to reduce insecurity among older people and to support their active engagement in life.**

## Background

Safety and security are considered basic human needs [1] and relevant domains constituting quality of life [2–4]. In terms of quality of life, the concept of safety is perceived as including notions such as security, personal control, competence and independence [4], and analogies are drawn between security, trust, confidence and certainty [5]. Thus, rather than merely being an expression of external factors related to safety, a more extensive construct views a sense of security as a multidimensional concept in which the inner experience is seen as theoretically related to a sense of coherence [4,6]. Indeed, prior studies among older people have suggested that a strong sense of security is associated with a tendency to view life as meaningful, with the ability to master life crises [6], and with having a sense of control [7], as well as trusting oneself [8,9]. However, a sense of security also includes an external dimension that is composed of elements such as feeling safe and secure in one’s surroundings and society and having trusting relationships and economic security [5,6,8,10].

Two previous distinct Scandinavian studies have investigated the topic of a global sense of insecurity among older people. Savikko et al. [11] studied older people residing in cities and rural areas in various parts of Finland and found that 9% felt insecure. Fagerström et al. [6] studied older people in the rural Bothnia region in both Finland and Sweden, and their study suggested that 10% felt insecure in their lives. In these studies, a sense of insecurity was associated with loneliness and dissatisfaction with one’s close relationships [11], as well as distrust in family, friends and neighbours [6]. Poor self-rated health (SRH) and decreased psychological well-being [11] as well as poor functional ability [6] were also associated with a sense of insecurity among older people. In a qualitative study, older Finnish people described their sense of insecurity as being related to feelings of loneliness, together with weakened health [12].

Apart from some qualitative studies shedding light on the matter [8,9,12], studies exploring a global sense of insecurity among older people are rare. Studies have mainly focused on external factors related to safety, such as fear of crime [10].

Yet, a desire for security is one of the main reasons why older people with disabilities or declining health decide to move into nursing homes [9,13,14]. Additionally, prior studies suggest that home care might not be sufficient to establish a sense of security [13]. One study compared a sense of security among those receiving old age care at home and those residing in nursing homes, and their results suggested that those living in a nursing home found their lives to be more secure [7].

### Aims

As a high value is placed on older people being able to age at home in today’s society, studying factors related to a sense of insecurity among home-dwelling older people is crucial for understanding what constitutes a sense of insecurity and, ultimately, for planning interventions aimed at improving a sense of security among older people.

The objective of this study was to investigate the extent to which home-dwelling older people aged ⩾75 years perceive their life to be secure or insecure. In addition, we investigated which more narrowly defined determinants this broad insecurity is composed of in order to target possible interventions.

## Methods

### Participants

The Helsinki Aging Study, a closed cohort study ongoing since 1989, has examined the health and well-being of home-dwelling older people in Helsinki [15]. Data have been collected using postal questionnaires that have been mailed on a regular basis every 10 years since 1989 to home-dwelling older people living in Helsinki.

In this study, we utilised questionnaire data from the 2019 Helsinki Aging Study sample. Age cohorts were retrieved from the Finnish Population Information System, and a random sample of 600 people from each age cohort aged 75, 80, 85 and 90 and all people aged 95 and 100 or older living in Helsinki were invited to participate in the study (total *N*=2917). The response rate was 74%, based on estimates of the number of survey recipients who had died, moved away or been institutionalised between the last population census and the retrieval of the sample. The Helsinki University Hospital Ethics Committee approved the study design.

### Measures

#### Sense of insecurity

The respondents were asked about their sense of security or insecurity: ‘Do you find your life to be secure or insecure at this moment?’ (very secure/moderately secure/moderately insecure/very insecure/indecisive). Based on their answers, we categorised the respondents into two groups—secure (very and moderately secure) and insecure (very and moderately insecure)—and we omitted those who remained indecisive.

#### Demographics

The respondents’ age and sex were determined by their personal identity code. Based on their answers, we determined the respondents’ living situation (living alone/living with someone), level of income (good/moderate/poor) and education (<8 years/8–12 years/>12 years).

#### Health and physical functioning

Objective health status was ascertained using the Charlson Comorbidity Index [16], a weighted index that takes into account the number and seriousness of co-morbidities. We listed 19 common medical conditions in the questionnaire (yes/no). Additionally, respondents had a chance to elaborate if they had been diagnosed with any other long-term illness(es) not listed in the questionnaire. The Charlson Comorbidity Index was then calculated based on the responses. Subjective health status was determined by assessing SRH. We asked: ‘How is your state of health?’ with the response options: ‘I consider myself healthy/moderately healthy/moderately unhealthy/unhealthy’. From the responses, we formed two categories: good SRH (healthy/moderately healthy) and poor SRH (moderately unhealthy/unhealthy) [17,18].

We assessed the respondents’ physical functioning by asking: ‘Does your general state of health allow you to walk outdoors easily?’ (yes/no, I need an assistive device/no, I need the help of another person/no, I cannot walk outside). The respondents were then categorised into two groups: those able to walk outdoors independently (yes) and those unable to do so (other options). We evaluated the respondents’ subjective functional ability by asking: ‘How would you rate your ability to function or your general physical condition at the moment?’ (very good/good/average/poor/very poor). From the responses, we formed three categories: good physical functioning (very good/good), average and poor (very poor/poor). We also asked: ‘Have you fallen in the last year?’ (yes, several times/yes, once or twice/no). From the responses, we formed two categories: those who had fallen, and those who had not.

The respondents were asked whether they had visited the emergency department in the last year (yes/no) and whether they have a general practitioner (GP) who they visit on a regular basis (yes/no).

#### Psychological well-being, social circumstances and active engagement

We inquired about feelings of loneliness: ‘Do you suffer from loneliness?’ (rarely or never/sometimes/often or always) and categorised respondents into those feeling lonely (sometimes/often or always) and those not feeling lonely (rarely or never). In addition, we asked: ‘How happy or unhappy do you feel at the moment?’ The answers generated two categories: happy (very happy/quite happy) and other (quite unhappy/very unhappy/indecisive).

To assess the respondents’ active social engagement, we asked: ‘Do you meet your friends at least weekly?’ (yes/no) and ‘Do you have living children?’ (yes/no). In terms of staying connected, we asked: ‘Do you use a smartphone?’ (yes/no) and ‘Do you use the Internet?’ (yes/no).

#### Perceptions of societal treatment

To evaluate the respondents’ perceptions of the societal treatment of older people, we asked: ‘How are older people treated in Finland in your opinion?’ (well/moderately/poorly). We formed two categories based on their answers: good treatment (well/moderately) and poor treatment (poorly).

### Statistical analysis

The descriptive statistics were presented as means with standard deviation (SD) or counts with percentages. Statistical comparisons between the secure and insecure groups were made using the *t*-test or chi-square test. To assess the importance of single variables on a sense of insecurity, we used a multivariable iterative stepwise forward selection and backwards elimination method for our analysis (probability for entry ⩽0.05, probability for removal ⩾0.1). We decided before the analysis which of the variables included in our questionnaire would be important determinants or confounders to test in the model. We introduced all variables from Table I into the model: age, sex, living alone, good income, walks outdoors independently, education, good SRH, Charlson Comorbidity Index, self-rated functional ability, has fallen during the past year, lonely, has a GP, has visited emergency department during the past year, has living children, meets friends at least weekly, perceives poor societal treatment, uses smartphone, and uses Internet. Stata v16.1 (StataCorp LP, College Station, TX) was used for the analysis.

**Table I.** Characteristics of the participants presented according to their sense of security.

Variable	Insecure, *N*=123	Secure, *N*=1396	*p*-Value
**Demographic characteristics**			
Women, *n* (%)	85 (69)	892 (64)	0.25
Age, *n* (%)			0.044
75–80	53 (43)	701 (50)	
85–90	48 (39)	545 (39)	
95+	22 (18)	150 (11)	
Living alone, *n* (%)	91 (76)	774 (57)	<0.001
Education, *n* (%)			
<8 years	39 (33)	318 (23)	0.02
8–12 years	58 (49)	699 (51)	
>12 years	21 (18)	365 (26)	
Good income, *n* (%)	35 (29)	532 (38)	0.038
**Health and functional ability**			
Charlson Comorbidity Index,^a^ mean (SD)	2.0 (1.7)	1.6 (1.6)	0.011
Good self-rated health, *n* (%)	72 (60)	1176 (85)	<0.001
Walks outdoors independently, *n* (%)	55 (45)	919 (66)	<0.001
Self-rated functional ability, *n* (%)			<0.001
Good	34 (28)	646 (47)	
Moderate	45 (37)	573 (42)	
Poor	43 (35)	161 (12)	
Has fallen during the past year, *n* (%)	62 (52)	517 (38)	0.003
Has a GP to visit regularly, *n* (%)	49 (42)	624 (46)	0.37
Has visited emergency department during the past year, *n* (%)	52 (44)	467 (34)	0.029
**Psychological well-being, social relationships and engagement**			
Lonely, *n* (%)	71 (58)	339 (25)	<0.001
Feels happy, *n* (%)	69 (57)	1214 (88)	<0.001
Has living children, *n* (%)	81 (66)	1154 (83)	<0.001
Meets friends at least weekly, *n* (%)	93 (77)	1229 (89)	<0.001
Uses smartphone, *n* (%)	34 (29)	596 (44)	0.002
Uses Internet, *n* (%)	50 (46)	694 (58)	0.02
**Perceptions of treatment**			
Perceives poor societal treatment, *n* (%)	39 (33)	247 (18)	<0.001

a Charlson et al. [16].

## Results

There were 1693 responses to the index item of insecurity. We omitted from the analyses those who remained indecisive and focused on those taking a stand, amounting to 1519 people altogether. Of all respondents, 7% (*n*=123) considered their lives insecure, whereas 83% (*n*=1396) felt secure. Table I presents the characteristics of the respondents. Of those feeling insecure, 69% were women, whereas the proportion of women among those feeling secure totalled 64%. The oldest age group was slightly overrepresented among those feeling insecure compared to those feeling secure. Of those feeling insecure, a larger proportion lived alone (76%) than those feeling secure (57%). Those feeling secure reported a higher income and had a higher education.

The Charlson Comorbidity Index was higher and SRH poorer among those feeling insecure than those feeling secure. Moreover, it was less common for those who felt insecure to be able to walk outdoors independently. The respondents’ subjective functional ability was lower among those who felt insecure than those who did not. Those feeling insecure were more likely to have fallen at least once in the past year, as well as more likely to have paid a visit to the emergency department, compared to those feeling secure. There was no difference between the groups in terms of having a GP to visit regularly.

Of those feeling insecure, 58% felt lonely compared to only 25% in the secure group. Among the insecure, fewer respondents felt happy compared to those in the secure group (57% vs. 88%). The insecure were also less likely to meet friends regularly or to have living children compared to the secure. Using a smartphone was more common among those who felt secure than those who reported feeling insecure, as was using the Internet.

Perceived poor societal treatment of older people was more common among those who reported feeling insecure than those who felt secure.

To assess the importance of single variables on a sense of insecurity, we used a multivariable iterative stepwise forward selection and backwards elimination method. The results are presented in Table II. It showed that loneliness (odds ratio (OR)=2.87; 95% confidence interval (CI) 1.76–4.67), perceived poor societal treatment of older people (OR=1.83, 95% CI 1.09–3.05) and living alone (OR=1.83, 95% CI 1.02–3.27) were associated with higher odds of insecurity. Having living children (OR=0.38, 95% CI 0.23–0.63), good SRH (OR=0.47, 95% CI 0.28–0.78) and meeting friends at least weekly (OR=0.54, 95% CI 0.3–0.96) were associated with lower odds of insecurity. Age, sex, income, education, co-morbidities, physical functioning, previous falls, paying regular visits to a GP, having visited the emergency department or using the Internet or a smartphone were not associated with a sense of insecurity.

**Table II.** Stepwise logistic regression analysis of associations with a sense of insecurity.

Variable	Odds ratio	95% confidence interval	*p*-Value
Female sex	1.36	0.8–2.34	0.26
Age (years)			
85–90	0.81	0.48–1.37	0.43
95+	1.19	0.59–2.4	0.62
Lonely	2.87	1.76–4.67	<0.001
Has living children	0.38	0.23–0.63	<0.001
Good SRH	0.47	0.28–0.78	0.004
Perceives poor societal treatment	1.83	1.09–3.05	0.02
Meets friends at least weekly	0.54	0.3–0.96	0.04
Living alone	1.83	1.02–3.27	0.04

Variables introduced into the model were: age, sex, living alone, good income, walks outdoors independently, education, good SRH, Charlson Comorbidity Index, self-rated functional ability, has fallen during the past year, lonely, has a GP to visit regularly, has visited emergency department during the past year, has living children, meets friends at least weekly, perceives poor societal treatment, uses smartphone, and uses Internet.

SRH: self-rated health.

## Discussion

Seven per cent of respondents reported feeling insecure in their lives. In bivariate analyses, those feeling insecure were older, more often living alone and living on a lower income than those feeling secure. They also had poorer health, functional ability and well-being. They had fallen more often or visited the emergency department during the past year compared to their secure counterparts. In the stepwise logistic regression analysis, however, only loneliness, living alone and perceived poor societal treatment of older people associated with insecurity, while having good SRH, having children who were living and meeting friends at least weekly associated with a lower sense of insecurity.

The strength of this study lies in its large representative sample of home-dwelling older people, of whom almost 50% were >85 years of age. The items used in our questionnaire have been used in the Helsinki Aging Study for 30 years [15]. They are easy for older people to understand, and the well-being items, for example, show good predictive validity [18]. Our comprehensive questionnaire enabled us to collect data on a broad set of variables, and we included all the variables that we considered possible determinants or confounders of a sense of insecurity.

The weakness of this study is that it was cross-sectional in nature, making it impossible to assess causality in our findings. Our single question item on insecurity poses another limitation, as the question itself does not account for the various dimensions of insecurity. Our aim, however, was to determine which of the variables in our study are determinants of a global sense of insecurity. Another weakness is that there may be selection bias, since those in better condition might have been more likely to respond to our questionnaire. Thus, the results can truly be generalised into home dwellers, who on average have better health and functioning than those living in assisted living or other long-term care facilities. In addition, due to selection bias, the prevalence of insecurity in our study may be an underestimate of the true prevalence, as only those in better condition might have responded. However, the 7% prevalence of a self-reported sense of insecurity in our study was in line with findings in those few previous studies investigating this topic [6,11]. Another limitation affecting the generalisability of our results is that it does not involve an environmental dimension, coupled with the fact that all of our participants resided in the same large city. The population distribution is uneven in Finland, as there are extensive rural areas that are sparsely populated, and most inhabitants live in large cities in the south [19]. Prior studies have shown that a person’s living environment plays an important role in their sense of safety. Living in a more deprived area with poorer access to local amenities and perceiving one’s neighbourhood as unsafe are associated with a sense of insecurity and loneliness [20–22]. However, in Finland, the distances between social classes are smaller than in many countries [23], and hence the differences between neighbourhoods in terms of safety are relatively small. Another environmental dimension missing in our study is the effect of season, as our questionnaires were sent in June and August. Wintertime in Finland is cold, and roads become slippery, which might increase older people’s sense of insecurity, and thus answers to our insecurity item might have been different had the questionnaires been sent during the winter months.

Consistent with prior studies, a sense of insecurity in our study was associated with poor SRH, loneliness, living alone and not having living children [7,11,24]. Our study suggests that meeting friends at least weekly is associated with a lower sense of insecurity. Several prior studies have suggested that there is a relationship between a sense of security and secure human relationships [6–8,24,25].

A sense of insecurity was associated in our study with perceived poor societal treatment of older people. A Finnish qualitative study suggested that older people felt that their insecurity was partly due to not being valued or to receiving insufficient help from society [12]. De Donder et al. [10] found that perceived ageism was associated with feeling unsafe in terms of external threats. Furthermore, a French study suggested that the activation of negative stereotypes may have detrimental effects on older people’s self-evaluation and functional ability and might lead to dependency and help-seeking behaviours among older people [26].

Our findings in the multivariable analysis suggest that a sense of insecurity is strongly associated with negative subjective experiences such as loneliness, poor SRH and perceived poor societal treatment of older people, whereas demographic factors such as age, sex and income seem to play a less significant role. Furthermore, besides SRH, other indicators of health, such as co-morbidities, physical functioning, previous falls, having a GP to visit regularly and visits to the emergency department all lost their significance in the multivariable analysis. Fagerström et al. [6] suggested in their study that lower physical functioning was related to a sense of insecurity, but the association was weak.

Prior studies have indeed suggested that a sense of security is a feeling within an individual [8], and that it originates from having self-confidence [8,9], a sense of control [7] and a sense of mastery [6] and from viewing life as meaningful [6]. The only objective factors associated with insecurity in our multivariable analysis were those related to a person’s social relationships, as those living alone, not meeting friends regularly and not having living children were at greater risk of feeling insecure. Those feeling socially isolated and not having children to rely on might regard their social safety network as weak, which could lead to a sense of insecurity. As suggested by Savikko [11], loneliness, social isolation and a sense of insecurity are distinct concepts but closely interrelated.

These findings have important implications when planning care and interventions aimed at improving older people’s sense of security. Many of our tested variables cannot be intervened by policy interventions. However, loneliness, for example, can be alleviated through interventions, as has been shown in our previous study [27], and it would be even more important now amidst the global COVID-19 pandemic [28]. If the prior studies concerning the need to establish security as one of the leading reasons for older people to move into nursing homes [9,13,14] hold true, the interventions to support security are of utmost importance and should be targeted at enabling social engagement and supporting social relationships. Moreover, it is vital to acknowledge that perceived poor societal treatment of older people was associated with a sense of insecurity. Recognising the perception of poor societal treatment and combating ageism [29] at the societal level is essential in promoting a sense of security in old age.

## Conclusions

According to our results, those suffering from loneliness, having a limited social network and experiencing poor societal treatment are at greater risk of feeling insecure. Our findings highlight the importance of recognising and combating loneliness in reducing insecurity among older people. In addition, society’s role in providing assistance and support for older citizens should not be underestimated, not forgetting the importance of combating ageism at the societal level.
